# New sex-determination system in the genus  *Panstrongylus* (Hemiptera: Reduviidae) revealed by chromosomal analysis of *Panstrongylus lutzi*

**DOI:** 10.1186/s13071-016-1574-6

**Published:** 2016-05-21

**Authors:** Silvia Menezes dos Santos, Silvia das Graças Pompolo, Teresa Cristina Monte Gonçalves, Simone Patricia Carneiro de Freitas, Elizabeth Ferreira Rangel, Jacenir Reis dos Santos-Mallet

**Affiliations:** Laboratório Interdiciplinar de Vigilância Entomológica em Diptera e Hemiptera, Instituto Oswaldo Cruz - Fiocruz, Av. Brasil 4365, 21045-900 Rio de Janeiro, RJ Brazil; Departamento de Biologia Animal, Laboratório de Citogenética de Insetos, Universidade Federal de Viçosa, Avenida PH Rolfs, s/n Campus Universitário, 36570-000 Viçosa, MG Brazil

**Keywords:** Cytogenetics, Karyosystematics, Triatominae

## Abstract

**Background:**

*Panstrongylus lutzi* (Neiva & Pinto, 1923) is a triatomine species native to Caatinga habitats in north-eastern Brazil. It is considered an important vector of Chagas disease in this region, presenting high rates of natural infection with *Trypanosoma cruzi* Chagas, 1909, and readily invading houses by flight. This study describes a previously unknown chromosomal sex system in the genus *Panstrongylus* based on *P. lutzi*.

**Methods:**

Fifth-instar and male adults of *P. lutzi* originating from municipality of Várzea Alegre, Ceará (Brazil) were analysed. Chromosomal analyses of male meiotic process were done by Giemsa staining.

**Results:**

Chromosomal analyses of male meiosis reveal a diploid chromosome number of 24 chromosomes (20 autosomes plus X1X2X3Y). During meiotic prophase I, the sex chromosomes remained close together, forming four heteropycnotic chromocenters in zygotene, and a single chromocenter in pachytene and diplotene. Still at the diplotene stage, each one of the ten autosomal bivalents showed an evident chiasma. In metaphase I, the four sex chromosomes appeared clearly separated. The three X chromosomes were the smallest of the complement and isopycnotic with respect to the Y chromosome. Two bivalents appear larger, whereas the other eight showed no significant difference in size.

**Conclusion:**

Karyotype analysis of *P. lutzi* revealed a new sex system in the genus *Panstrongylus*. This result is of utmost importance to karyosystematics of *P. lutzi*, and demonstrates the need for further studies of this type in the subfamily Triatominae.

## Background

Subfamily Triatominae includes more than 150 living species and two fossils [1]. All are capable of transmitting the parasite *Trypanosoma cruzi* Chagas, 1909, the etiological agent of Chagas disease. Most species are considered of little to no epidemiological importance, described as either secondary or occasional vectors [2]. *Panstrongylus lutzi* is a species restricted to the Caatinga biome in north-eastern Brazil, and participates in the enzootic cycle of this disease. It has high rates of natural infection with *T. ​cruzi*, and readily invades houses by flight [3, 4].

The Hemiptera are cytogenetically characterized by presenting holocentric chromosomes, where the centromere is diffusely distributed [5, 6]. Cytogenetic techniques and analysis of chromosome behaviour during meiosis have provided characters for species differentiation [7–12] and determination of interspecific variation [8, 13–16], and have contributed to studies of evolutionary relationships [9, 17, 18]. Several characters such as chromosome number, system of sex determination, and the comparative size of autosomes have been proven useful for differentiating triatomine species.

Karyotypic studies of the Triatominae were initiated using the karyotype description of *Triatoma sanguisuga* [19]. In 1950, cytogenetic studies were resumed and new karyotypes were described in the literature [20]. Ueshima (1966) [6] initiated cytotaxonomy in triatomines by describing the diploid chromosome set of twenty new species, proposing that 22 chromosomes (20A + XY) is the type number for the Triatominae and emphasizing the importance of cytogenetic studies for the taxonomy of these vectors. Currently, 88 species (ranging from 21 to 25 chromosomes) have described karyotypes [21].

Cytogenetic analysis of the genus *Panstrongylus* was initiated in 1950 with the karyotype description of *Panstrongylus megistus* [20]. Crossa et al. [14] conducted an important cytotaxonomic study of genus *Panstrongylus*, characterizing six species: *Panstrogylus chinai*, *Panstrongylus rufotuberculatus*, *Panstrongylus lignarius*, *Panstrongylus geniculatus*, *Panstrongylus tupynambai* and *P. megistus*. However, there are 14 living *Panstrongylus* species [1] and an understanding of the cytogenetic characteristics of the other eight species is of extreme importance for describing taxonomy and evolutionary relationships in this group of vectors.

To increase knowledge of chromosomal behaviour in the genus *Panstrongylus*, we characterized for the first time the meiotic cycle of *P. lutzi* using a conventional staining technique.

## Methods

Twenty male adults and 20 fifth-instar *P. lutzi* nymphs were analyzed. Sex was determined by genital plate morphology. The insects originated in the municipality of Várzea Alegre, Ceará, and were kept in colonies in the Interdisciplinary Laboratory for Entomological Vigilance of Diptera and Hemiptera, Department of Medical and Forensic Entomology, Oswaldo Cruz Institute, Rio de Janeiro, Brazil.

Insects were dissected and testes removed using Ringer's solution. Testes were then mounted on slides using methodology adapted from Imai et al. [22]; this is to our knowledge the first use of these methods in a non-hymenopteran insect. Briefly, the material is transferred to a histological slide which is flooded with fixative I (4:3:3 water:ethanol:acetic acid). The slides are then transferred to a stereomicroscope and two drops of fixative I are added, initiating separation. Before the material is completely dry, two drops of fixative II (1:1 ethanol:acetic acid) are added, followed by two drops of fixative III (100 % acetic acid). Pieces of filter paper were placed on the edges of the slides to remove excess fixative solution. Slides were then dried at room temperature for 24 h and stained with Giemsa diluted in Soerensen buffer (pH 6.8, 1:30 dilution) for five minutes at room temperature.

Meiotic cells were observed and images captured using an Olympus BX 60 microscope with a 100× objective, equipped with a Q Color 3 Olympus® image capture system.

## Results

**Fig. 1:**
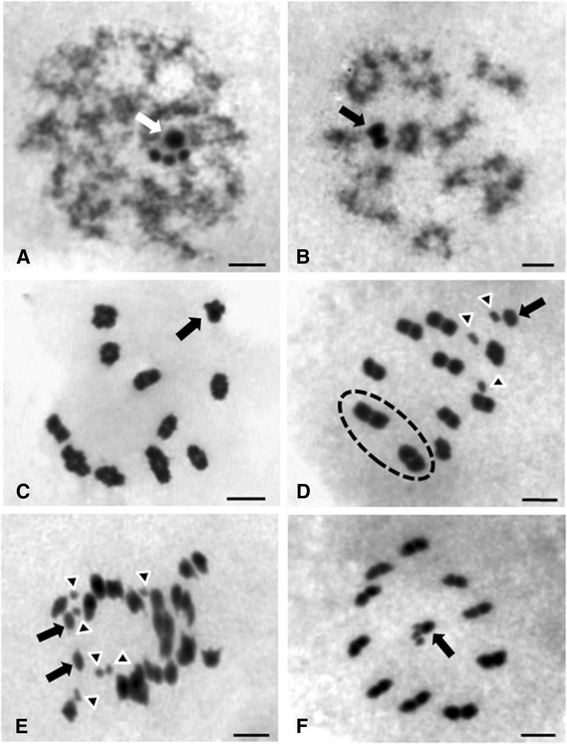
Meiosis in *Panstrongylus lutzi* males (Giemsa staining). 2n = 20A+ X_1_X_2_X_3_Y **a** Zygotene with four heteropycnotic chromocenters (*arrow*). **b** Pachytene with a single chromocenter formed by sex chromosomes (*arrow*). **c** Late Diplotene. Each one of the ten bivalents showed one chiasma while the sex chromosomes appeared close associated (*arrow*) **d** Metaphase I showing ten bivalents, three X chromosomes (X_1_X_2_X_3_) (*arrowheads*) and Y chromosome (*arrow*). The dashed circle indicates two major bivalents. **e** Anaphase I. Equational division of sex chromosomes indicating X_1_X_2_X_3_ chromosomes (*arrowheads*) and Y chromosome (*arrow*). **f** Metaphase II with ten half-bivalents and the four sex chromosomes forming a pseudotetravalent (*arrow*). *Scale-bars*: 5 μm

Analysis of male meiotic metaphases revealed a diploid chromosome number of 24 chromosomes, including 20 autosomes and four sex chromosomes (X1X2X3Y). During the first meiotic prophase, the sex chromosomes were kept close together forming four heteropycnotic chromocenters in zygotene (Fig. 1a) or as a single chromocenter in pachytene and diplotene (Fig. 1b, c). Still at the diplotene stage, each one of the ten bivalents showed an evident chiasma (Fig. 1c). In metaphase I, four sex chromosomes showed to be separated: three X chromosomes of similar size were the smallest of chromosome complement and appeared to be isopycnotic regarding Y chromosome (Fig. 1d). The autosomes showed two largest bivalents, whereas the others had no significant differences in size. In anaphase I, the sex chromosomes exhibited equational segregation while the autosomes showed reductional segregation (Fig. 1e). In metaphase II (Fig. 1f), the four sex chromosomes were located in the centre of the metaphase plate.

## Discussion

The analysis of cytogenetic characteristics supports the hypothesis that there are distinct chromosomal lines within the Triatominae.

However, many monophyletic groups present the same cytogenetic characteristics, as in the phyllosoma complex [9] and in brasiliensis, rubrovaria and matogrossensis subcomplexes [18]. Furthermore, similarity of chromosomal characteristics among species in the tribe Rhodniini suggests a monophyletic origin [16], as well as the fact that all species have the same number of chromosomes (22) and the same system of sex determination (XY) [17].

The Triatomini tribe presents an extensive variation in several chromosomal characters supporting the existence of different evolutionary lines, with widely varying degrees of chromosomal differentiation depending on the species group considered. This group presents three different sex systems in males: XY, X1X2Y and X1X2X3Y. In the genus  *Panstrongylus*, all species studied to date showed only X1X2Y sex system [14]. In this paper we included a new sex system of *P. lutzi* (X1X2X3Y) differing from those previously described within this genus. The variation in the number of sex chromosomes in *P. lutzi* can be explained by fragmentation of the original X chromosome [6], or as resulting from other chromosomal rearrangements [23]. Cytogenetic analyses with other banding techniques could provide valuable information about the origin of the additional X chromosome in *P. lutzi*.

Molecular analyses and experimental hybridization among *P. lutzi* and other *Panstrongylus* spp. are needed to assess the evolutionary relationships of this genus.

## Conclusion

Karyotypic analysis of *P. lutzi* revealed a new sex system in the genus *Panstrongylus*. This result is of utmost importance for karyosystematics in triatomines in order to clarify the taxonomic identification and chromosome evolution within this insect group.
